# The effects of low-toxic herbicide Roundup and glyphosate on mitochondria

**DOI:** 10.17179/excli2021-4478

**Published:** 2022-01-10

**Authors:** Olha M. Strilbyska, Sviatoslav A. Tsiumpala, Ivanna I. Kozachyshyn, Tetiana Strutynska, Nadia Burdyliuk, Volodymyr I. Lushchak, Oleh Lushchak

**Affiliations:** 1 Department of Biochemistry and Biotechnology, Vasyl Stefanyk Precarpathian National University, 57 Shevchenko Str., Ivano-Frankivsk, 76018, Ukraine; 2 Research and Development University, 13a Shota Rustaveli Str., Ivano-Frankivsk, 76000, Ukraine

**Keywords:** roundup, glyphosate, mitochondria, oxidative stress

## Abstract

The effects of pesticides on the health of non-target living organisms in agricultural areas are critically important aspects for their safe use. Their release into the environment is an inevitable aspect for predicting and evaluation of the risk of their application. Roundup, a glyphosate-based herbicide, has been designed as an effective pesticide against weeds and now is the most widely used agrochemicals around the world due to its highly specific action of the biosynthesis of certain amino acids in plants. Despite it is claimed to be low toxic for not-target organisms, due to its broad application Roundup and products of its degradation were detected in organisms of diverse animals and humans. In this review, we describe animal and human studies of general adverse effects of Roundup and its principal substance glyphosate with focus on endocrine disruption, oxidative stress and behavioral disorders. At mechanistic level, we focus on the potential toxicity of the herbicide Roundup and glyphosate as effectors of bioenergetic functions of mitochondria. Their effects on mitochondrial membrane potential and oxidative phosphorylation are among described to date critical components responsible for its toxicity. Finally, we discuss general molecular mechanisms potentially involved in the interaction between glyphosate and mitochondria which to some extent are associated with generation of reactive oxygen species.

## Abbreviations

AMPA - aminomethylphosphonic acid; ROS - reactive oxygen species; TCA - tricarboxylic acid cycle

## Introduction

Using pesticides to increase crop production and improve human health can be of great benefit to society. In pesticide development, these benefits are targeted at specific pests, but there is also a need to assess the potential impact on non-target organisms including humans (Annett et al., 2014[4]; Damalas and Eleftherohorinos, 2011[18]; Lushchak et al., 2018[52]). 

Roundup is the most commonly used pesticide in the world (Duke, 2020[22]). Since its appearance on the market, its use has been increased enormously. Its global use reached 825,804 metric tons in 2014 (Benbrook, 2016[10]). Roundup Original® contains active ingredient glyphosate, N-(phosphonomethyl) glycine, in the form of its isopropylamine salt, as well as surfactant polyethoxylated tallow amine, whereas Roundup Transorb R® contains the potassium salt of glyphosate with surfactants. Both contain different compositions of so-called "inert" ingredients, which are believed to aere more toxic than glyphosate (Rissoli et al., 2016[72]). Roundup has relatively short half-life in water up to 91 days and soil up to 197 days which is affected by environmental conditions (Miller et al., 2010[58]). Due to their cumulative properties, they circulate in ecosystems and accumulate in non-target organisms and can be included in food chains (Bai and Ogbourne, 2016[7]), showing significant toxicity to a wide range of organisms (Lushchak et al., 2018[52]). Glyphosate residues were detected in the urine of farmers who live in the roundup treated areas suggesting potential impact of Roundup on the humans (Rendon-von Osten and Dzul-Caamal, 2017[71]). Moreover, Roundup components are proven to present in rivers (0.1-0.7 mg/l), sediments (0.0-4.9 mg/kg) and soil (0.5-4.3 mg/kg), sometimes even at concentrations close to toxic (Peruzzo et al., 2008[68]).

Investigation of potential toxicity of Roundup (Samsel and Seneff, 2015[74]) and its major active substance glyphosate on various model organisms may help to elucidate the role of defense mechanisms in its toxic effects. Evaluation of the impact of Roundup and its components on metabolism, cell signaling, apoptosis and aging with particular attention to mitochondria in order to understand interference with bioenergetic functions is very important from theoretical and practical points of view. In this review, we will highlight established to date and molecular mechanisms of effects of Roundup and its components on respiratory chain and oxidative phosphorylation in the mitochondria. 

## Impact of the Glyphosate and Glyphosate-Based Herbicides on Living Organisms

### Absorption, accumulation, transformation and release of glyphosate in the organism

Glyphosate can enter the human body and be excreted in unchanged form (Hove-Jensen et al., 2014[39]). The bioaccumulation factor for glyphosate varies significantly depending on species, time of exposure and concentration of the acting compound. Some components of the formulation of glyphosate-based herbicides enhance entering of glyphosate into organisms (Contardo-Jara et al., 2009[17]). For example, macroinvertebrates namely *Chironomids *and *Gastropoda* could accumulate glyphosate in their body from the bathing area of Lake Lednica up to 10.2 µg kg^-1^ (Rzymski et al., 2013[73]). Glyphosate was found in different tissues of slaughtered cows including the intestine, liver, muscle, spleen and kidney at a concentration of 20 ng kg^-1^ with no significant influence of glyphosate residuals on animal (Krueger et al., 2014[46]). Approximately 35-40 % of glyphosate at concentration 10 mg/kg was absorbed from the gastrointestinal tract of Sprague-Dawley rats (Brewster et al., 1991[14]). Within 7 hours after oral glyphosate administration, almost 40 % of the absorbed material was eliminated with the urine, about 50 % was associated with the small intestine and after 7 days nearly all of the absorbed material was eliminated from the organism. The authors suggested that urinary and fecal pathways of excretion are the main routes for glyphosate excretion (Brewster et al., 1991[14]). The European Commission (2002[26]) informed that glyphosate can absorb rapidly and to small extent (~30 %) and be rather quickly excreted from animal body; glyphosate metabolism in animals was found to be very limited and the effects of glyphosate on the body occur when the organism is exposed for a relatively long period. 

While glyphosate is a small molecule it can penetrate passively through the cell membrane. For example, glyphosate at low concentration can penetrate the epithelial barrier of human epithelial cells only to small extent. However, exposure to higher concentrations of glyphosate (10 mg/ml) reduced the transepithelial electrical resistance and increased permeability of mannitol into epithelial cells (Vasiluk et al., 2005[82]). 

### Metabolism of glyphosate in the intestine bacterial community 

Glyphosate was patented as an antimicrobial agent and was detected in steadily increasing amounts in the genetically modified Roundup-Ready corn and soy feed of cows, pigs, chickens, farmed shrimp and fish as well as it is ubiquitous in the Western diet of humans (William, 2010[84]).

**Figure 1:**
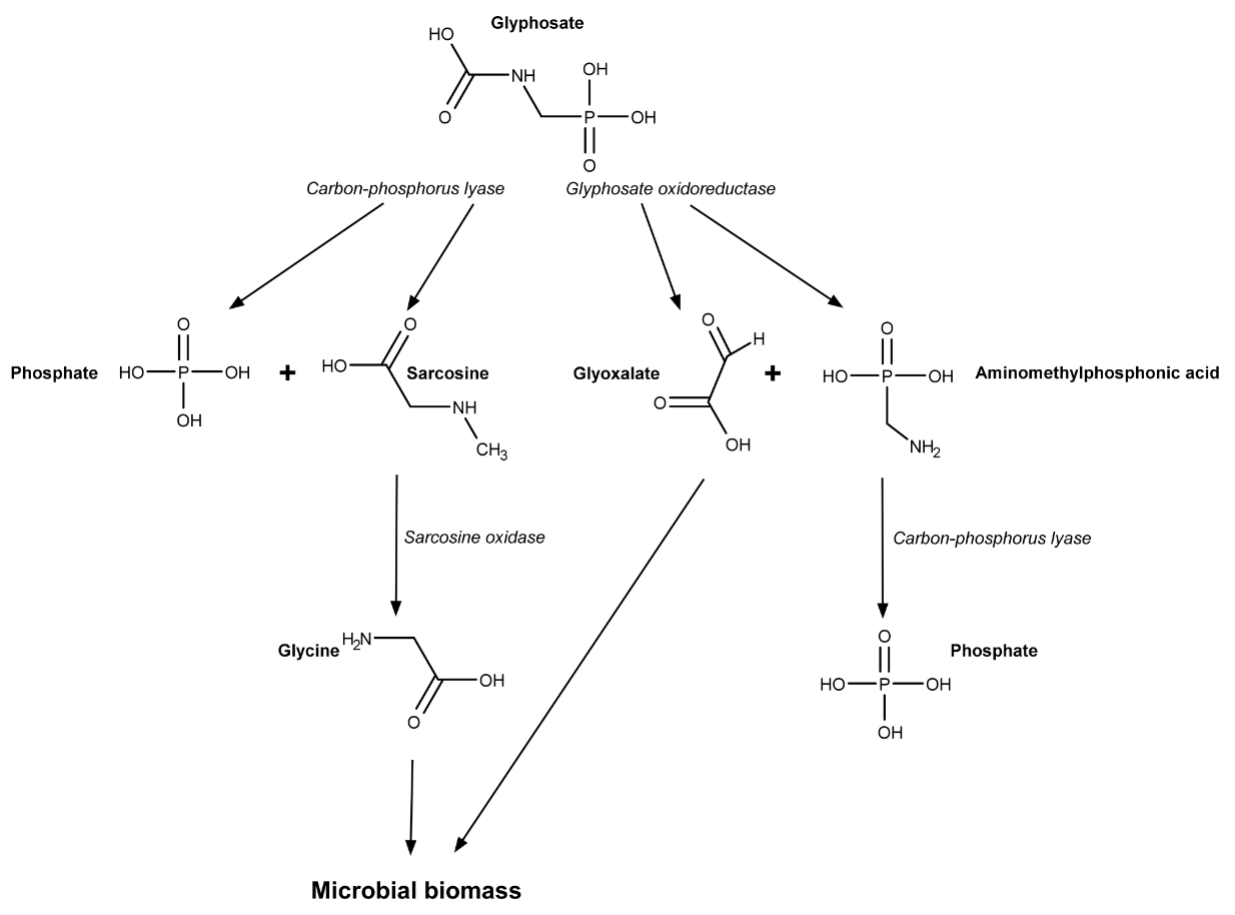
Metabolism of glyphosate in bacteria

Glyphosate affects the commensal bacterial community in the animal intestine (Nielsen et al., 2018[60]). Moreover, some amount of glyphosate is degraded primarily by intestine microorganisms (Mesnage and Antoniou, 2020[55]). There are at least two ways of glyphosate metabolism in bacteria. The first, use of glyphosate as a sole source of phosphorus was found in *Pseudomonas aeruginosa*. These bacteria is one of a small number of resistant bacterial species with the ability to metabolize glyphosate, a feature that might be exploited for soil remediation (Abdel-Megeed et al., 2013[1]). Carbon-phosphorus lyase (C-P lyase) catalyzes the first step of glyphosate metabolism replacing the phosphonate group from glyphosate by H2O molecule to form sarcosine (Figure 1(Fig. 1)). Next, sarcosine dehydrogenase catalyzes sarcosine conversion to glycine which, in turn, further may be converted to formaldehyde (Kishore and Jacob, 1987[43]; Shinabarger and Braymer, 1986[75]). 

The second pathway of bacterial glypho-sate metabolism was found in *Escherichia coli, Arthrobacter sp. *and* Pseudomonas sp.* This pathway occurs in the bacteria that actively use phosphorus to produce energy. These microorganisms convert glyphosate to aminomethylphosphonic acid (AMPA) with glyphosate dehydrogenase. Next, AMPA is cleaved to methylamine by C-P lyase. Finally, methylamine dehydrogenase catalyzes methylamine breakdown into formaldehyde and ammonia (Hove-Jensen et al., 2014[39]). Intermediates and reduced coenzymes at above described transformations are used to produce ATP.

Cytochromes P450 are a superfamily of enzymes containing heme as a cofactor and functioning as monooxygenases. They are known to be involved in the metabolism of xenobiotics (Esteves et al., 2021[25]) including glyphosate. Exposure of rats to sublethal glyphosate doses decreased cytochrome P450 levels in the liver (Larsen et al., 2014[49]). 

A product of glyphosate degradation phosphonate AMPA demonstrated toxic effects on humans at a concentration of 0.25 mM (Wang et al., 2016[83]). Treatment by AMPA enhanced generation of reactive oxygen species (ROS) and resulted in higher level of methemoglobin in human erythro-cytes (Kwiatkowska et al., 2014[48]). Import-antly, AMPA is less toxic as compared to glyphosate. Both AMPA and glyphosate exhibited genotoxicity for European eel (*Anguilla anguilla* L.) at concentrations 11.8 µg L^-1^ and 23.6 µg L^-1^, however, AMPA did not induce marked DNA oxidation (Guilherme et al., 2012[33], 2014[34]).

The European Food Safety Authority (2002[26]) reported that degradation of glyphosate to AMPA in the human body is very limited. The bioavailability of glyphosate after oral administration was 23.21 % and only 6.49 % of it was metabolized to AMPA (Anadón et al., 2009[2]). Both, glyphosate and AMPA, were found in the urine at concentrations of 0.28±0.38 and 0.30±0.33 µg/L, respectively. However, glyphosate and AMPA were not found in human milk and in this way is supposed not to be dangerous for children (McGuire et al., 2016[54]). Therefore, it can be concluded that glyphosate is not the only precursor of AMPA in the environment. Indeed, several phosphonates can also degrade to AMPA (Huntscha et al., 2018[40]).

## Molecular Mechanisms of Interaction between Glyphosate and Mitochondria

### Impact of glyphosate on key bioenergetic enzymes and levels of intermediates of tricarboxylic acid cycle 

The activity of lactate dehydrogenase (LDH) is used as a low specific marker of cellular damage due to exposure to pesticides (Jurisic et al., 2015[41]; Klein et al., 2020[44]). The activity of LDH indicates about switching of anaerobic glycolysis to aerobic respiration. The additional function of the enzyme is the involvement in the protective mechanisms via contributing to DNA repairing processes, maintaining lactate and pyruvate homeostasis and modulation of redox potential (Lemire et al., 2008[50]). The activity of LDH in the serum of human was significantly affected by glyphosate treatment (El-Demerdash et al., 2001[23]). Glyphosate exposure decreased LDH activity in the human brain (Olorunsogo, 1990[63]; Cattani et al., 2014[16]). The activities of glucose-6-phosphate dehydrogenases (G6PDH) and malate dehydrogenases (MDH) were affected at glyphosate exposure of rats to glyphosate (Daruich et al., 2001[19]). 

Succinate dehydrogenase (SDH) is a membrane-bound enzyme linking metabolism and aerobic energy production (Kumari, 2018[47]). This enzyme couples the oxidation of succinate to fumarate in the Krebs cycle with the reduction of ubiquinone to ubiquinol. Due to hydrophilicity and structural similarity between glyphosate and succinate (Ugarte, 2014[81]), glyphosate attaches to the binding site of succinate (Burchfield et al., 2019[15]). The electron microscopy analysis showed reduced respiratory activity of mitochondria under Roundup treatment of liver hepatocytes (Malatesta et al., 2008[53]). The activity of SDH, which is a key component of the TCA cycle and the respiratory chain, is used as a biomarker to evaluate Roundup cytotoxicity in human cells (Mesnage et al., 2015[57]). 

In addition, glyphosate acts as a trigger to the pentose phosphate pathway (PPP), which is involved in the generation of reducing equivalents in NADPH form (De Freitas-Silva et al., 2017[20]). Enhancement of PPP activity also reflects induction of oxidative stress within the cell (Tang, 2019[79]).

### Influence on mitochondrial membrane and membrane potential

Mitochondria generate chemical energy in a form of adenosine triphosphate (ATP) used in many biochemical reactions within the cell. The functional shift in mitochondria can lead to severe alterations of the general metabolism. In 2005 Peixoto showed that rat liver mitochondria were negatively affected by a glyphosate-based pesticide in combination with other compounds (Peixoto, 2005[66]).

The mitochondrial membrane potential is an essential component in the regulatory mechanism of respiratory rate, ATP synthesis and ROS generation. Protein complexes of the respiratory chain in mitochondria are essential components for maintaining the electrochemical potential of hydrogen ions needed to synthesize ATP. The shifts in the respiratory chain function may result in alterations in mitochondrial membrane potential (Zorova et. al., 2018[86]). Hence, membrane permeability is believed to be an indicator of Roundup and glyphosate-based herbicides toxicity. Glyphosate induced hyperpolarization of the mitochondrial membrane in the mitochondria from brain of zebrafish *Danio rerio* (Pereira et al., 2018[67]). Electron microscopy of *Cyprinus carpio* treated with Roundup revealed myelin-like structures in hepatocytes, mitochondria swel-ling and mitochondrial internal membrane disruption (Szarek et al., 2000[78]). However, glyphosate exposure at a concentration of 10 mg/kg and roundup at a concentration of 10 mM for either 4 and 24 hours had no significant effect on mitochondrial membrane integrity in the *Substantia nigra* in male Wistar rats (Astiz et al., 2009[6]). Glyphosate up to 15 mM did not affect mitochondrial membrane potential, but Roundup at a concentration of 10 mM disrupted mitochondrial membrane potential in preparations from liver of Wistar rats (Peixoto, 2005[66]). The mitochondrial mem-brane potential was lower in rat heart H9c2 cells exposed to a mixture of glyphosate and surfactant TN-20 (Kim et al., 2013[42]). Roundup also decreased mitochondrial potential in rat hepatoma tissue culture (Malatesta et al., 2008[53]). Glyphosate disturbed mitochondrial membrane potential in the immortalized human HaCaT cell line (Heu et al., 2012[37]). Glyphosate-based herbicide TouchDown (TD) at a range of concentrations 3 % to 10 % decreased proton transmembrane gradient in *E. coli* (Burchfield et al., 2019[15]).

Glyphosate-based herbicides can also modify mitochondrial membrane. Disruption of mitochondrial membrane potential is associated with high levels of ROS and can correlate with activation of caspases which can be harmful to the cell (Olorunsogo et al., 1980[64]). 

### Mitochondrial respiratory chain inhibition

The electron transport chain (ETC) is the last step of glucose metabolism associated with energy production in ATP form. It consists of a set of protein complexes in the inner membrane of the mitochondria. Electrons from NADH or FADH2 pass through a series of electron transporters to oxygen producing water (Kumari, 2018[47]). To test the effect of Roundup and glyphosate on the bioenergetic functions of mitochondria, numerous experiments were performed. In particular, the investigation of isolated mitochondria of rat liver demonstrated chelating properties of glyphosate. Indeed, glyphosate can bind Fe^3+^, Fe^2+^, Cu^2+^, Zn^2+^, Mn^2+^, Ca^2+^ and Mg^2+^ in a small amount (Harris et al., 2012[36]). The chelating properties of glyphosate may partially explain its reduced energetic efficiency of mitochondrial respiratory chain. 

Glyphosate inhibited the energy-linked function by 46 % in the mitochondria isolated from rat liver (Olorunsogo et al., 1979[65]) and retarded NAD+/NADH converting process in liver cell bу 34.5 % in albino rats (Olorunsogo et al., 1980[64]). It was demonstrated that glyphosate at concentrations up to 5 mM had no effects on respiratory chain and ratio ADP/O. However, 0.5 mM of Roundup significantly depressed the respiratory chain and ADP/O ratio. Moreover, Roundup at concentrations up to 15 mM depressed operation of complex ІІІ and uncoupled respiration rates (Peixoto, 2005[66]). 

**Figure 2:**
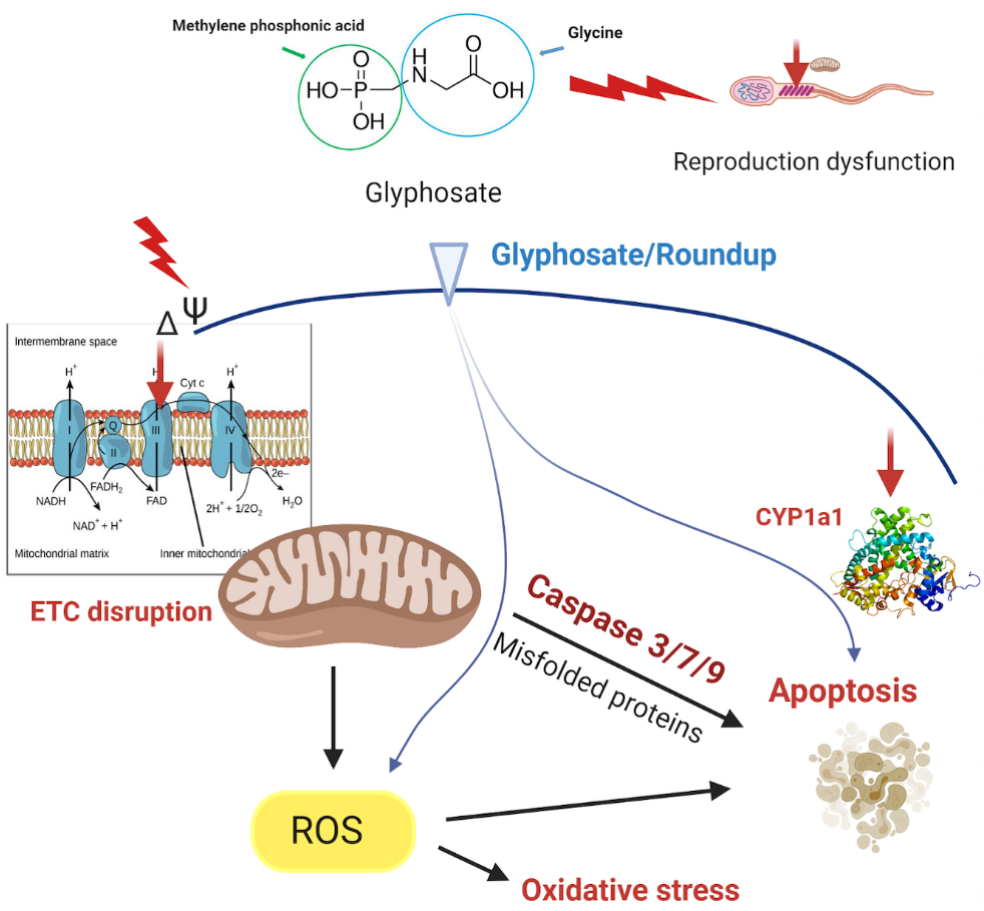
Effects of glyphosate and Roundup related to mitochondria

Roundup is known to disrupt mitocho-ndrial bioenergetic reactions (Figure 2(Fig. 2)). Alterations in membrane potential (∆Ψm) and mitochondrial respiration are the classical parameters to analyze basic mitochondrial functions (Zorova et. al., 2018[86]). Despite glyphosate led to higher mitochondrial membrane permeability to protons and Ca^2+^, it is not able to act like a protonophore (Olorunsogo, 1990[63]). Furthermore, the carboxylic group in the molecule of glyphosate has a similar pKa value (5.6) to the acetic acid pKa value (4.76). Acetic acid at pH 7.1 may transport protons across the membrane. However, the glyphosate mole-cule could not participate in the transmembrane transport of protons, since at pH 7.1 it is mainly charged. Thereby, glyphosate can penetrate via the lipid membrane only in a small amount. 

Exposure of *Caenorhabditis elegans* to glyphosate at the concentrations of 5.5 % and 9.8 % inhibited the respiratory chain in mitochondria (Bailey et al., 2018[8]). Exposure of in *Escherichia coli* to TouchDown at the concentrations from 3 % to 10 % decreased oxygen consumption, complex II activity and relative ATP levels, but increased complex IV activity (Burchfield et al., 2019[15]). Glyphosate at the concentrations of 0.065 and 1.0 mg/L inhibited NADH dehydrogenase and cytochrome c oxidase. Zebrafish exposure to glyphosate lowered transcript levels of the genes *ndufa6, sdhc *and* cox1* which encoded components of the mitochondrial respiratory chain (Pereira et al., 2018[67]).

Roundup reduced the efficiency of the electron transport chain via inhibition of succinate dehydrogenase and succinate cytochrome c reductase (Peixoto, 2005[66]). Roundup affected the redox electron transport chain at the level of complexes II and III (Peixoto, 2005[66]). It also exhibited cytotoxicity to the human embryonic kidney cell line due to the suppression of the respiratory activity of mitochondria (Mesnage et al., 2013[56]). The observed alterations in mitochondrial bioenergetics caused by Roundup cannot be exclusively attributed to glyphosate as the principal active ingredient, but either by other components of the formulations or even possible synergy between them. Moreover, the reduced energetic efficiency of mitochondria may be a result of toxic effects from the impairment of the energy requirements of the cell and the crucial importance of energy metabolism. 

### Mitochondrial dysfunction and production of reactive oxygen species 

Growing evidence suggests that organismal exposure to commercial herbicide formulations may induce oxidative stress and inhibit mitochondrial respiratory chain (Bailey et al., 2018[8]). The molecular mechanisms induction of oxidative stress induction by glyphosate and glyphosate-based herbicides are well characterized. Uncoupling of mitochondrial oxidative phosphorylation may be a major effect of glyphosate and glyphosate-based herbicides intoxication (Olorunsogo et al., 1979[65]; Peixoto, 2005[66]; Pereira et al., 2018[67]). The impaired mitochondrial function caused by glyphosate-based herbicides can be related to increased ROS generation (Bailey et al., 2018[8]; Gomes and Juneau, 2016[31]). Moreover, glyphosate or glyphosate-based herbicides exposure resulted in the alteration of the brain antioxidant system activity (Astiz et al., 2009[6][5]; Bali et al., 2019[9]; Cattani et al., 2014[16]; Gallegos et al., 2020[28]). In order to assess oxidative stress parameters and mitochondrial inhibition by the herbicide treatment *in vivo*, the nematode *Caenorhabditis elegans* was exposed chronically (24 h) to various concentrations of the glyphosate-containing herbicide TD. Following TD exposure, the function of specific mitochondrial electron transport chain complexes was evaluated. Animal *in vivo *exposure to mid- and high-TD concentrations lead to inhibition of oxygen consumption by the isolated mitochondrial fractions in *C. elegans* (Bailey et al., 2018[8]).

In addition, while glyphosate increased the permeability of the inner mitochondrial membrane for protons and Ca^2+^, it may induce oxidative stress itself or in its formulations *in vivo* (El-Shenawy, 2009[24]; Gehin et al., 2006[30]; Olorunsogo, 1990[63]). Roundup opened voltage-dependent calcium channels and endoplasmic reticulum receptors (such as IP3 and ryanodine), which caused an increase in intracellular Ca^2+^ concentration (De Liz Oliveira Cavalli et al., 2013[21]; Peixoto, 2005[66]). Indeed, Ca^2+^ is considered to be a key player to increase mitochondrial ROS levels due to induction of structural changes in the inner mitochondrial membrane (Kowaltowski and Vercesi, 1999[45]). Furthermore, inhibition of complex I and IV increased mitochondrial ROS production (Bolter and Chefurka, 1990[13]; Sipos et al., 2002[76]). Ca^2+ ^can also influence operation of the mitochondrial respiratory chain (Kowaltowski and Vercesi, 1999[45]).

Consequently, ROS-induced oxidative damage to mitochondrial components could be a reason for membrane potential disruption, which leads to dysregulation of cell function and cell death (De Liz Oliveira Cavalli et al., 2013[21]). 

### Involvement of apoptosis 

Apoptosis, a programmed cell death, is a highly regulated process. Two main pathways are known to trigger apoptosis: the first, the intrinsic pathway which is mediated by mitochondria and the second, the extrinsic pathway mediated by death receptors FASR, TNFR1, TRAIL R1/R2. The intrinsic pathway is also called the mitochondrial pathway and involves the release of cytochrome c from the mitochondria under cellular stress (Nirmala and Lopus, 2020[61]). Apoptosis involves also caspases, a family of cysteine proteases (Boatright and Salvesen, 2003[12]).

Roundup and glyphosate-based herbici-des may affect the activity of caspases and induce apoptosis (Figure 2(Fig. 2)). Exposure of rat heart H9c2 cells to a mixture of either 5 mM or 10 mM of glyphosate and 2.5 mM surfactant TN-20, increased the activity of caspases 3/7 and 9, but glyphosate alone did not affect the activities (Kim et al., 2013[42]). In the hepatoma cell line HepG2, caspases 3/7 were activated after 24 and 48 hours of Roundup exposure (Gasnier et al., 2009[29]). Two hours exposure to Roundup at concentrations 75, 100, 125 µg/ml increased the activity of caspase-9 and at concentrations 100-125 µg/ml increased the activity of caspase-3 in the human alveolar carcinoma A549 cell line which was proposed to be related to cytotoxicity through DNA damage (Hao et al., 2019[35]). It has been demonstrated that exposure to glyphosate at a concentration of 40 mM for 24 hours increased the amount of apoptotic nuclei within the cell and activated autophagic pathway in neuronal differentiated PC12 cells (Gui et al., 2012[32]). A higher mRNA level of caspase-3 was demonstrated under 500 mg/kg glyphosate treatment of male Sprague Dawley rats (Tang et al., 2017[80]). 

### Activity of mitochondria in the sperm cell

The importance of mitochondrial energetics is connected with hyperactivated motility of sperm and the phenomenon of sperm capacitation (Piomboni et al., 2012[69]). Furthermore, mitochondria are the main ROS source here and are involved in biosynthesis of steroid hormones and regulation of steroid receptor functions (Psarra and Sekeris, 2008[70]). Therefore, sperm mitochondrial functionality is an important indicator of gamete function and reproductive toxicology.

Many studies have focused on the effects of glyphosate and glyphosate-based herbici-des on sperm quality. Effects varied according to species, type of herbicide and range of herbicide concentrations. Exposure to glyphosate at the concentrations 5 mg/L and 10 mg/L for 24 hours and 96 hours reduced mitochondrial bioenergetics by 20 % and 35 % in sperm cells of zebrafish *Danio rerio* (Lopes et al., 2014[51]). Treatment with Roundup at a concentration of 1 mg/L reduced the mitochondrial staining in human sperm cells (Anifandis et al., 2017[3]). Mitochondrial dysfunction caused by Roundup treatment was associated with lower mitochondrial membrane potential and resulted in the progressive reduction of sperm motility (Anifandis et al., 2017[3]). Glyphosate negatively affected mitochondrial respiration efficiency in human sperm cells (Ferramosca et al., 2021[27]). Thus, glyphosate and glyphosate-based herbicides can harm the activity of mitochondria in the sperm cell that causes a threatening impact on reproductive function. Using the pig as a model, it was demonstrated that Roundup is more toxic than pure glyphosate itself, even at equivalent concentrations of glyphosate (Nerozzi et al., 2020[59]). 

The impaired mitochondrial activity under Glyphosate/Roundup treatment may decrease ATP generation and/or shift in the redox balance. These events significantly impact sperm cell motility and plasma membrane stability (Nerozzi et al., 2020[59]). An increase in mitochondrial-dependent apoptosis is another additional consequence of mitochondrial dysfunction under Roundup exposure (Anifandis et al., 2017[3]). Now it is clear, that glyphosate and glyphosate-based herbicides may have detrimental effects on fertilizing ability.

## Toxicity of "Inert" Ingredients of Roundup

Studies regarding the effects of other ingredients of Roundup formulations are mostly limited to the evaluation of surfactant used to increase glyphosate bioavailability (Williams et al., 2000[85]). Polyethoxylated tallow amine (POEA) is the predominant surfactant used in Roundup formulations worldwide. Early studies of Birch (1977[11]) showed that acute toxicity of POEA is higher than of Roundup formulation. LD50 for oral dose in rats was reported as 1200 mg/kg and dermal toxicity in rabbits was found at the dose of 1260 mg/kg. Based on U.S. EPA POEA falls into second-least-toxic category (III) an thus is considered as these considerations, POEA is considered to be only “slightly” toxic and does not represent an acute toxicity hazard. Later the toxicity of POEA were extendiverly evaluated in rats. POEA in doses higher that 1500 ppm have induced wight gain decrease and inflammation (Ogrowsky, 1989[62]), as well as intestinal irritation, decreased food consumption, body weight gain, and some alterations in serum hematology and clinical parameters (Stout, 1990[77]). Despite significant maternal toxicity in pregrant rats there were no effects observed in offspring providing evidence that POEA is not teratogenic or developmental toxin in rats (Holson, 1990[38]).

No-Observed-Adverse-Effect Levels (NOAEL), and Margins of Exposure (MOE) for POEA in human beings were developed based on maternal toxicity in the rat developmental toxicity study. The lowest NOAEL of 15 mg/kg/day was selected as a reference point for risk assessment purposes giving NOES 577 and 461 in children and adults, respectively. However, calculation of MOE for children based on a NOAEL for maternal toxicity is not biologically relevant and thus MOE of 1380 was estimated using the NOAEL of 36 mg/kg/day from the subchronic rat study. 

The potential risks to humans were determined for pesticide applicators, as highest potential group for exposure among adults, and farm children age 1 to 6 years because they receive the highest dietary intake of all subpopulations. Chronic aggregate exposure in children was calculated to be 26 µg/kg/day and 32.5 µg/kg/day in adults. The ingestion of food residues accounted for all of the exposure in children, while dermal/inhalation exposure resulting from spraying of formulation was the predominant way contributing to exposure of applicators. Estimates of aggregated acute exposure in adult applicators was 163 µg/kg a day and children 9.11 µg/kg/day). The acute oral LD50 of POEA is approximately 1200 mg/kg. The estimated acute exposure values are significatly lower than this value. 

## Conclusions and Perspectives

This review provides evidence of Roundup and glyphosate-based herbicides can impact non-target living organisms. Both, glyphosate and Roundup, appear to act as organismal toxicants, detrimentally affect cell function and survival. Having a wide range of effects on metabolism, cell signaling, apoptosis, dysfunction of gametogenesis and aging, Roundup and its components appear to act through disruption of bioenergetic functions of mitochondria. Alterations in the mitochondrial bioenergetic reactivity have drastic consequences on cellular function through perturbation of the bioenergetic charge and balance of the cell. Disruption of mitochondrial membrane potential is associated with high levels of reactive oxygen species and can be correlated with activation of caspases, which is harmful to a cell due to the high risk of apoptosis. Detrimental consequences of glyphosate and Roundup affect mitochondrial functionality that impairs sperm parameters may have a deleterious effect on fertilizing ability. Hence, mitochondria are supposed to be good biomarkers of glyphosate toxicity. Given the growing concern over potential reproductive effects of Roundup, the current research provides valuable mechanistic information for their environmental risk assessment. Further investigation of molecular mechanisms of negative effects of Roundup and glyphosate-based herbicides on the operation of mitochondria in non-target organisms, standardization of conditions to testing may provide reliable biomarkers to access quantitative parameters of their toxicity. The developed approaches to test the harmful effects of glyphosate-based herbicides may be extended to evaluation of detrimental effects of other pesticides.

## Notes

Sviatoslav A. Tsiumpala and Ivanna I. Kozachyshyn contributed equally.

Volodymyr I. Lushchak and Oleh Lushchak (Department of Biochemistry and Biotechnology, Vasyl Stefanyk Precarpathian National University, 57 Shevchenko Str., Ivano-Frankivsk, 76018, Ukraine; E-mail: oleh.lushchak@pnu.edu.ua) contributed equally as corresponding authors.

## Declaration

### Funding

This work was partially supported by the grant from National Research Foundation of Ukraine #2020.02/0270.

### Conflict of interest

The authors declare that they have no conflict of interest.
